# The Prevalence of and Risk Factors Associated with Musculoskeletal Disorders among Sonographers in Central China: A Cross-Sectional Study

**DOI:** 10.1371/journal.pone.0163903

**Published:** 2016-10-03

**Authors:** Qingmin Feng, Shenglin Liu, Lei Yang, Mingxing Xie, Qiang Zhang

**Affiliations:** 1 Department of Medical Engineering, Union Hospital, Tongji Medical College, Huazhong University of Science and Technology, 1277 JieFang Avenue, Wuhan, 430022, China; 2 Healthcare Ergonomics Lab, Union Hospital, Tongji Medical College, Huazhong University of Science and Technology, 1277 JieFang Avenue, Wuhan, 430022, China; 3 Department of Occupational and Environmental Health, Tongji Medical College, Huazhong University of Science and Technology, 13 HangKong Road, Wuhan, 430030, China; 4 Department of Medical Ultrasound, Union Hospital, Tongji Medical College, Huazhong University of Science and Technology, 1277 JieFang Avenue, Wuhan, 430022, China; Rush University Medical Center, UNITED STATES

## Abstract

**Objective:**

Studies from industrialized countries show that musculoskeletal disorders (MSD) occur commonly in sonographers. However, little is known about sonographers in China, where the awareness of ergonomics and MSD, workload, and available equipment/facilities may differ. We aimed to investigate the prevalence of MSD and associated risk factors in sonographers in central China.

**Methods:**

A cross-sectional survey was conducted with 381 sonographers from 14 randomly selected tertiary hospitals in Hubei province, central China. Musculoskeletal symptoms (using the Nordic Questionnaire) and risk factors (mostly derived from the Health Benefit Trust survey instrument and the Dutch Musculoskeletal Questionnaire) were recorded. Multivariate logistic regression was used to quantify associations between risk factors and MSD.

**Results:**

The 12-month period prevalence of MSD was 98.3%, being highest in the neck (93.5%) and shoulder (92.2%), followed by the lower back (83.2%), wrist/hand, upper back, and elbow. Factors contributing to neck pain were psychological fatigue, shoulder abduction and trunk bend-and-twist posture. Height-adjustable tables and chairs were protective factors. Shoulder pain was associated with female sex, health status, mental stress, shoulder abduction, and trunk bend-and-twist posture. Height-adjustable chairs and the awareness of adjusting the workstation before scanning were protective factors. Elbow pain was associated with health status and height-adjustable tables. Wrist/hand pain was associated with female sex, bending the wrist, and working with obese patients. Upper back pain was associated with shoulder abduction, height-adjustable chairs, and device location. Lower back pain was associated with the number of scans performed per day, awkward postures, bending the trunk, twisting or bending the neck forward, and using a footrest.

**Conclusions:**

This study suggests a high prevalence of MSD in sonographers in central China. Hence, it is necessary to improve the awareness of MSD by training, and the ergonomics of their current work environment by addressing physical workload, and psychological and equipment/facility-related factors.

## Introduction

Musculoskeletal disorders (MSD) are one of the most important occupational health issues in healthcare workers [1], particularly among sonographers [2]. Numerous workplace exposures and demographic and psychological risk factors have been shown to be linked to MSD, such as prolonged static muscle activity, repetitive movements, poor posture, physical conditioning, and other factors, including age, sex, and work stress [3–7]. MSD affect working life, reduce work productivity, increase absence due to ill health, and can result in chronic occupational disability [8]. A few studies have reported a high prevalence of MSD among health professionals in China, such as dentists, nurses, surgeons, and physicians, with prevalence ranging from 67.5–88.0% [9–14]. However, little attention has been paid to occupational health hazards in the sonography profession in China. International studies on MSD in sonographers have been conducted in Canada, the USA, and Europe. These studies reported a prevalence ranging from 63–98%, and revealed consistently high frequencies worldwide of shoulder, neck, wrist, and back disorders A comprehensive survey by Pike et al. [15], conducted throughout Canada and the USA, revealed that 81% of the sampled sonographers experienced MSD while scanning, with the neck (75%), shoulders (74%), and wrists (65%) identified as the most affected body regions. Evans et al. [16] conducted a comparative study with a higher number of respondents, and found that shoulder pain (75%) was the most common complaint, with older and more experienced sonographers reporting finger, hand, and wrist pain more frequently.

While scanning, sonographers usually hold uncomfortable static postures for long periods [17–18]; this can lead to chronic muscular fatigue or pain. Long working hours and the number of scans performed can also aggravate the pain, and the intense schedule may increase the psychological strain of high mental stress and fatigue in workplace, which may also potentially affect workers’ health [19]. The workload of sonographers in China is very heavy owing to the shortage of medical staff, unevenly distributed medical resources, the large population base, and population aging [20], especially in tertiary hospitals due to daily visits per physician of 7.9[21]. All of the situations mentioned above increase the number of scans and working hours of Chinese sonographers. Furthermore, about 75 percent of ultrasonic diagnostic equipment in tertiary hospitals has been imported from western countries [22], which might not conform to Chinese people’s characteristics, since the average height of Chinese people is 167.1 cm for males and 155.8 cm for females [23]. The use of high-tech equipment without effective ergonomic design and workplace training might result in more MSD [24]. Potential lack of ergonomics (for example the adjustability of tables, screens, keyboards, chairs, or control panels) in the workplace may also increase the risk of MSD.

In Western countries, attention to and awareness of MSD in the sonography profession has increased due to the large number of reported musculoskeletal symptoms and training programs. Training on the prevention of MSD in ultrasonic scanning has been conducted among sonographers in the United Kingdom; a successful educational program on the awareness of ergonomics and overall risks of MSD might be helpful in reducing MSD [25]. However, MSD have not yet been incorporated into the occupational disease reporting system in China and, to our knowledge, little evidence is available regarding the prevalence of MSD in Chinese sonographers.

The purpose of this study was to investigate the prevalence and severity of work-related MSD among sonographers working in tertiary hospitals in Hubei province, central China, and to identify the associated risk factors for MSD in various body locations.

## Materials and Methods

### Participants

To obtain information on prevalence and risk factors of MSD, we conducted a cross-sectional survey of all sonographers working in randomly selected 14 tertiary hospitals in Hubei province of central China in March 2015. The study was planned to be completed within four months from March 1, 2015 to July 1, 2015.

Based on sample size calculations, the random and representative sample size required would be 219 sonographers using an estimated 80% prevalence of MSD [26] and allowing a 5% tolerable error, based on a 95% confidence interval (CI). There are ~2,000 registered sonographers working in the 71 tertiary hospitals in Hubei province. Considering the data collection quality and availability, the study randomly selected 14 hospitals from all the 71 hospitals, to avoid questionnaire delivery and collection difficulties if all 2,000 sonographers were randomly sampled.

We posted 381 paper-based questionnaires and introductions to all on-duty sonographers working in the ultrasound departments of the 14 selected hospitals in March 2015. Three follow-up phone calls were made to the departments’ directors to encourage participation in the subsequent four months. This study was approved by the Ethics Committee of Tongji Medical College, Huazhong University of Science and Technology (IORG No: IORG0003571) prior to commencing the study. Each questionnaire had attached an informative letter on the first page, which clarified the free participation, anonymity, and confidentiality of this study. We did not ask participants for written consent. Those who answered the questionnaire were deemed to have provided voluntary informed consent to participate. This procedure was specifically approved by the ethics committee of Tongji Medical College, Huazhong University of Science and Technology.

All sonographers with at least one year of work experience and without trauma injury or accidents were invited to participate in the study, and required to complete and return the questionnaires within four weeks.

### Questionnaire

A paper-based questionnaire was designed, based on a review of the literature, established and validated questionnaires [15–16, 27–28], field observation, and personal interviews with experienced sonographers. The questionnaire included information on demographic and psychological factors, work scheduling and tasks, work-related postures, and equipment and facilities. The outcomes of the risk of musculoskeletal symptoms were ascertained.

The final version of the questionnaire consisted of 5 sections. The first section included demographic questions about age, sex, height, weight, body mass index (BMI), handedness, education, accumulated exercise duration per week, and type of exercise performed. BMI was calculated by the formula weight/height squared (kg/m^2^) [29] and classified into two groups (normal: <24 kg/m^2^ and overweight: >24 kg/m^2^) [29]. Psychosocial factors in the first section included current health status (good, average, or bad), job satisfaction (satisfied or dissatisfied), the level of psychological fatigue (high, medium, or low) and mental stress (high or low) in the work place.

The second section assessed work scheduling and tasks factors including work years, scan years, scan hours per week, number of scans per day, average time per scan, times and duration of rest during the work day, types of scans performed and the percentage of each type.

Sections three and four dealt with working postures, and equipment and facilities. Working postures were derived from the standardized Dutch Musculoskeletal Questionnaire (DMQ) [27] and modified by Yang Lei et al. [30], including awkward posture adopted when performing scan tasks (such as neck, wrist, and trunk twisting or bending, and shoulder abduction). Working postures were measured using a dichotomous scale (No/Yes). Participants were asked whether they were often exposed to the awkward work postures mentioned in the questionnaire in their work without further explanations or definitions upon the exposure variables, so the assessment of risk factors was qualitative in this study. The working postures section has been validated by previous study of Du Weiwei et al. [31]. Working equipment and facilities section focused on the ergonomics of equipment and facilities, including the frequently used and preferred machines, equipment maneuverability and adjustability, chair and table adjustability, work place changing frequency, and the awareness of adjusting the workstation before starting work. Questions pertaining to preferred improvements to current workstation and environment were included.

The fifth section was a modified version of the Standardized Nordic questionnaire [28], which was amended and contextualized by previous studies according to a cross-cultural understanding of Chinese people [30], and has been used and validated by previous national studies [31–34]. The participants were asked whether they had experienced pain in the neck, shoulder, elbow, wrist/hand, upper back, or lower back lasting more than 24 hours in the past twelve months. If any positive symptoms occurred, supplementary questions around musculoskeletal symptom frequency, severity, and impact on work needed to be answered.

Ten pilot tests were distributed to professional sonographers working in one hospital before the questionnaire was posed to all the sample hospitals. Although no major problems with the questionnaire were identified, minor changes were made to improve the instrument design based on the ten participants’ comments and feedback.

### Data analysis

Data were entered to EpiData version 3.1 and exported to SPSS® statistical software (version 21, SPSS Inc., Chicago, IL, USA) for analysis. Descriptive statistics were used to show frequency distributions, percentages, mean ± standard deviation, and range for all responses. For the continuous variables, participants were divided into three groups using tertile boundaries.

Multivariate logistic regression was used to determine the effects of potential risk factors on MSD symptoms with crude and adjusted odds ratios (OR)—adjusted for sex, age, and education level. Potential predictors of MSD were selected by univariate logistic regression at a p-value of <0.05, and entered to the binary logistic regression models along with age, sex, and education level as potential confounding factors. A Spearman correlation matrix was used to diagnose collinearity in relationships (ρ > 0.6) between independent variables to avoid an unstable and inaccurate logistic regression model. As the variables ‘age’ and ‘work years’ showed significant correlations with ‘scan years’ (ρ > 0.6), we removed the variables ‘work years’ and ‘scan years’, and kept ‘age’ for the multivariate analysis. Significant correlation was also found between ‘work hours’ and ‘scan hours’ (ρ = 0.64); we included ‘scan hours’ in the multivariate analysis. A stepwise backwards procedure was used to develop the multivariate logistic regression model. The likelihood ratio statistic was used for variable entry (p < 0.05) and removal (p > 0.1). The analysis was performed separately for the neck, shoulder, elbow, wrist/hand, upper back, and lower back. Crude ORs with 95% confidence intervals (CI) at a significance level of 0.05 were used to determine associations between risk variables and musculoskeletal symptoms. Partially completed questionnaires, with over 20% missing data, were excluded from the analysis. Other missing items were defined as system-missing values by SPSS for analysis.

## Results

### Sample demographics and psychological factors

Altogether 381 questionnaires were posted, with a response rate of 66.4%. Of the 253 completed questionnaires, 21 were excluded because of repetitive answers, partially completed answers, work years of less than 12 months, or pain or discomfort caused by trauma or an accident. Finally, 232 responses were analyzed (S1 Appendix).

Table 1 describes the demographics and characteristics of the samples. Most of the participants for analysis were females (75.0%). The mean age was 33.1 ± 7.2 years. The mean height was 163.2 ± 6.8 cm, with 28.0% of participants being shorter than 160 cm. Job satisfaction was reported by 75.9% of respondents, while 24.1% were dissatisfied with their job. Psychological fatigue and mental stress at work were reported as high level by 25.4% and 68.1% of participants, respectively.

**Table 1:** Description of Demographic and Psychological Factors.

**Variables**	**N**	**Percentage**
***Sex***		
Female	174	75.0%
Male	58	25.0%
Missing	0	0.0%
***Age***		
<29 years	63	27.2%
29–34 years	97	41.8%
>34 years	67	28.9%
Missing	5	2.2%
***BMI***		
<24 kg/m^2^	182	78.4%
>24 kg/m^2^	37	15.9%
Missing	13	5.6%
***Handedness***		
Right hand	214	92.2%
Left hand	18	7.8%
Missing	0	0.0%
***Education***		
Graduate	137	59.1%
Bachelor	88	37.9%
Other	5	2.2%
Missing	2	0.9%
***Health status***		
Good	54	23.3%
Average	144	62.1%
Bad	34	14.7%
Missing	0	0.0%

### Work scheduling and tasks

The data on work scheduling and tasks are listed in Table 2. The average years spent scanning was 8.7, slightly longer than the years spent working in the current job (8.2 years). On average, sonographers worked 43.4 hours per week, and spent 37.5 hours per week performing scans. Of the respondents, 42.2% reported scanning more than 50 patients per day.

**Table 2:** Description of Work-Related Factors.

**Variables**	**N**	**Percentage**
***Scan years***		
<5 years	79	34.1%
5–10 years	84	36.2%
>10 years	69	29.7%
Missing	0	0.0%
***Scan hours per week***		
≤40 hours	170	73.3%
>40 hours	61	26.3%
Missing	1	0.4%
***Number of scans per day***		
<30 patients	33	14.2%
30–50 patients	101	43.5%
>50 patients	98	42.2%
Missing	0	0.0%
***Scan duration per patients***		
<10 minutes	167	72.0%
≥10 minutes	65	28.0%
Missing	0	0.0%
***Break times during workday***		
Over twice	33	14.2%
Once or twice	129	55.6%
No rest	70	30.2%
Missing	0	0.0%
***Night shift***		
One or more times per month	165	71.1%
Less than one time per month	61	26.3%
Missing	6	2.6%
***Scanning obese patients***		
Rarely	83	36.1%
Often	147	63.9%
Missing	2	0.9%

### Working posture

Overall, 86.6% of participants reported having to adopt awkward work postures while scanning. Participants reported often needing to bend (46.6%), twist (47.0%), or simultaneously bend and twist (72.4%) their trunk; to bend their neck forward (75.4%) or backward (26.3%), or twist their neck (49.6%) and hold the posture for a prolonged period; to maintain shoulder abduction (82.3%); to bend (85.3%) or twist (77.2%) the wrist and hold the position for a prolonged period; and to exert substantial force with their hands or arms (93.1%).

### Equipment, facility, and environment

The three most frequently used machines included two brands imported from Europe and America (76.8%) and one domestic brand (23.2%). When asked if the ultrasound machine screen was adjustable, 76.3% reported that it was, but 22.0% of these respondents had never adjusted the screen. The usual location of the ultrasound equipment was reported as mostly being directly in front of them (69.0%). The position of the screen was reported as being higher than eye level in 40.9% of responses.

In terms of the chairs and examination tables, 59.5% indicated having a height-adjustable chair to use for examinations, 71.6% had chairs with an armrest, 77.6% had chairs with a footrest, but 89.7% reported using nonadjustable tables. Only 22.4% had the awareness to adjust workstation before scanning in order to keep a relatively comfortable working posture.

When given possible options for improving the current workplace, 74.1% participants wanted to have appropriate armrests to support the arm/elbow to exert pinching and pushing force when scanning; 64.7% participants hoped to improve the equipment design, maneuverability, and adjustability to make it easier to use; 62.5% participants wanted to have an adjustable examining table; 59.5% participants indicated that a lighter probe and cable (or even no cable) were essential; and 42.7% participants wanted a height-adjustable chair.

### Musculoskeletal prevalence and symptoms

The self-reported period prevalence of MSD was defined as the prevalence of MSD in the past twelve months. Table 3 shows the number and percentage of the 12-month period prevalence, and its severity within different body sites. The 12-month period prevalence of MSD was 98.3%. Pain and discomfort were mostly found in the neck (93.5%), shoulder (92.2%), lower back (83.2%), and wrist/hand (79.3%), followed by the upper back (72.8%) and elbow (41.8%). The proportions of pain were significantly different between various body sites (p < 0.001).

**Table 3:** Pain Prevalence during the 12-Month Period and its Severity by Anatomical Location.

Regions	N	Percentage	Severe	Moderate	Slight
***Neck***	217	93.5%	13.4%	55.2%	25.0%
***Shoulder***	214	92.2%	18.5%	56.4%	17.2%
***Lower back***	193	83.2%	19.0%	50.0%	14.2%
***Wrist/hand***	185	79.7%	9.9%	47.8%	22.0%
***Upper back***	169	72.8%	9.4%	39.3%	23.1%
***Elbow***	97	41.8%	3.0%	20.7%	18.0%

Specific information about the symptoms of those who reported pain included description of the severity, frequency, impact on job duty, and medical diagnosis of the musculoskeletal discomfort(s). When asked to rate the severity of pain, most participants rated their pain as moderate. Of those who reported pain, 9.6% had changed their job duty, 15.8% had been absent from work due to the MSD in the preceding twelve months, and 58.3% reported that they had sought assistance from a doctor, physiotherapist, or massage therapist for their related pain in the past 12 months.

### Multivariate logistic regression

Tables 4–7 present the results of the multivariate logistic regression. For neck pain, the adjusted OR for psychological fatigue (medium level vs. low level, and high level vs. low level), trunk bend-and-twist posture, shoulder abduction, and decreased adjusted OR for height-adjustable tables and height-adjustable chairs were statistically significantly increased (Table 4). For the outcomes of shoulder pain, there were statistically significant increases in adjusted OR for female sex, health status (average vs. good), mental stress (high vs. low), shoulder abduction, and trunk bend-and-twist, and decreased adjusted OR for height-adjustable chairs and for adjusting the workstation before scanning (Table 5). In terms of elbow pain, there were significantly increased OR for health status (bad vs. good, OR 4.9, 95% CI 1.81–13.37) and height-adjustable tables (OR 2.8, 95% CI 1.04–7.63). Female sex (OR 2.8, 95% CI 1.36–6.06), wrist bending (OR 3.5, 95% CI 1.45–7.97), and scanning obese patients (OR 2.6, 95% CI 1.29–5.24) were significantly associated with increased odds of wrist pain in the adjusted analysis.

**Table 4:** Multivariate Analysis of MSD Involving the Neck.

**Variables**	**Specification**	**P**	**Crude OR [95% CI]**	**Adjusted OR [95% CI]**
***Psychological fatigue***	Low	0.017		
	Medium	**0.016**	7.6 [1.64, 35.58]	8.3 [1.49, 45.90]
	High	**0.045**	4.7 [0.99, 21.99]	7.0 [1.05, 46.97]
***Bend and twist trunk***	No			
	Yes	**0.008**	6.0 [1.98, 18.44]	6.4 [1.62, 25.59]
***Shoulder abduction***	No			
	Yes	**0.012**	4.7 [1.60, 13.85]	6.4 [1.51, 27.13]
***Height-adjustable table***	No			
	Yes	**0.016**	0.1 [0.04, 0.40]	0.2 [0.03, 0.70]
***Height-adjustable chair***	No			
	Yes	**0.008**	0.2 [0.05, 0.95]	0.1 [0.01, 0.50]

*Note*. OR = odds ratio; CI = confidence interval. Age, sex, education, exercise, number of scans per day, awkward posture, bend neck forward, exert force, and workplace change frequency had no effect in the final model.

**Table 5:** Multivariate Analysis of MSD Involving the Shoulder.

**Variables**	**Specification**	**P**	**Crude OR [95% CI]**	**Adjusted OR [95% CI]**
***Sex***	Male			
	Female	**0.025**	2.3 [0.83, 6.16]	4.7 [1.22, 17.88]
***Health status***	Good	0.079		
	Average	**0.024**	3.9 [1.44, 10.39]	4.2 [1.21, 14.40]
	Bad	0.997	-	-
***Mental stress***	Low			
	High	**0.002**	6.6 [2.27, 19.40]	10.1 [2.34, 43.44]
***Bend and twist trunk***	No			
	Yes	**0.038**	3.7 [1.39, 9.86]	4.1 [1.08, 15.38]
***Shoulder abduction***	No			
	Yes	**0.040**	3.4 [1.22,9.31]	4.3 [1.07, 17.61]
***Height-adjustable chair***	No			
	Yes	**0.004**	0.4 [0.13, 1.24]	0.1 [0.03, 0.51]
***Adjust workstation before scanning***	No			
	Yes	**0.002**	0.3 [0.12, 0.87]	0.1 [0.02, 0.42]

*Note*. OR = odds ratio; CI = confidence interval. Age, education, psychological fatigue, height-adjustable table, awkward posture, and exert force had no effect in the final model.

**Table 6:** Multivariate Analysis of MSD Involving the Upper Back.

**Variables**	**Specification**	**P**	**Crude OR [95% CI]**	**Adjusted OR [95% CI]**
***Shoulder abduction***	No			
	Yes	**0.011**	2.5 [1.26, 5.12]	2.8 [1.26, 6.13]
***Height-adjustable chair***	No			
	Yes	**0.001**	0.4 [0.21, 0.75]	0.3 [0.15, 0.63]
***Device located in front of sonographers***	No			
	Yes	**0.002**	0.3[0.16, 0.70]	0.3[0.13, 0.63]

*Note*. OR = odds ratio; CI = confidence interval. Age, sex, education, health status, and mental stress had no effect in the final model.

**Table 7:** Multivariate Analysis of MSD Involving the Lower Back.

**Variables**	**Specification**	**P**	**Crude OR [95% CI]**	**Adjusted OR [95% CI]**
***Number of scans per day***	<30	0.107		
	30–50	0.308	1.9 [0.77, 4.59]	1.8 [0.58, 5.58]
	>50	**0.041**	3.8 [1.42, 10.29]	3.6 [1.06, 12.16]
***Awkward posture***	No			
	Yes	**0.010**	4.1 [1.78, 9.32]	4.0 [1.39, 11.53]
***Bend trunk***	No			
	Yes	**0.047**	4.2 [1.82, 9.53]	2.7 [1.01, 6.97]
***Bend neck forward***	No			
	Yes	**0.004**	3.9 [1.88, 7.97]	3.8 [1.51, 9.39]
***Twist neck***	No			
	Yes	**0.005**	3.0 [1.40, 6.31]	3.9 [1.51, 9.85]
***Chair with footrest***	No			
	Yes	**0.019**	0.2 [0.07, 0.83]	0.2 [0.03, 0.74]

*Note*. OR = odds ratio; CI = confidence interval. Age, sex, education, health status, psychological fatigue, scan minutes, twist trunk, bend and twist trunk, shoulder abduction, exert force, and height adjustable table had no effect in the final model.

For the outcomes of upper back pain, there were significantly increased OR for shoulder abduction, and decreased odds for height-adjustable chairs and having the device located in front of the sonographer (Table 6). For the outcomes of lower back pain, the adjusted analysis revealed significantly increased OR for the number of patients scanned per day (>50 vs. <30), awkward postures, trunk bending, bending the neck forward, and twisting the neck, and a significantly decreased OR for using a chair with a footrest (Table 7).

## Discussion

To our knowledge, this is the first self-reported questionnaire survey concerning Chinese medical sonographers’ work-related musculoskeletal pain, associated risk factors, and the effect on daily activities. Participants were sampled from medical sonographers working in tertiary hospitals in Hubei province, central China. Work-related MSD were most frequently reported in the neck, shoulder, lower back, and wrist/hand regions. These findings were associated with sex, high mental stress and fatigue in the work place, number of scans performed per day, scanning obese patients, working postures, the adjustability of tables and chairs, as well as the awareness to adjust devices/facilities before scanning.

The final questionnaire response rate of 60.9% was comparable to or even higher than those reported in previous surveys [16, 25, 35–38]. The number of responses analyzed (n = 232) was slightly higher than the calculated number required (n = 219), based on a random selection formula. Thus, the sample can be considered representative of sonographers working in tertiary hospitals in Hubei province. Compared with the cross-sectional study conducted by Pike et al. [15] and Evans et al. [16], our study’s sample size was much smaller and only considered sonographers working in one level of hospital care. However, our study does, to some extent, reveal serious problems faced by Chinese sonographers, such as heavy psychological strain in the work place, physical workload, poor workplace ergonomics, and the lack of ergonomic awareness. We also found that sonographers in China commonly work for prolonged hours with insufficient rest periods, and perform a greater number of scans than sonographers in other countries. However, these factors showed no significant effect on the present of MSD in this study.

In this study, 98.3% of respondents reported scanning whilst in pain or discomfort in the past 12 months. The results were higher than most studies of MSD among medical sonographers (63.0–96.4%) [16, 35–36, 39–41], but similar to Feather’s report (98.7%) [42]. In keeping with other studies [15–16, 39], our study found the highest 12-month prevalence of MSD in the neck, shoulder, wrist/hand, elbow and back (both upper and lower back). However, the 12-month prevalence for each region was higher than in other studies. Considering the heavier workload of sonographers working in tertiary hospitals in China [21], the prevalence of this study may not be representative of sonographers working at other levels of health care.

MSD are also very common in other allied health professionals in China (e.g. dentists, nurses, physicians, or surgeons), among whom the prevalence of neck and shoulder pain is reported to be 42.3–83.8% and 33.6–73.5%, respectively [9–14]. Dentists, who also work in awkward static postures, have been found to have a comparable but lower prevalence of neck pain (83.8%) and shoulder pain (73.5%) in China [9]. Sonographers spend almost a whole workday in sitting positions when scanning. Studies have shown positive association between sitting time and neck-shoulder pain [43], as well as low back pain [44]. The prevalence of neck and shoulder pain in the current study was 93.5% and 92.2%, respectively. A relatively lower prevalence of neck (75.6%) and shoulder (77.3%) pain were also found among office workers with high workload computer use who also work for prolonged periods in sitting postures [45].Women in this study tended to be more likely to present with shoulder (OR 4.7) or wrist/hand (OR 2.8) pain than men. Sex differences in the presentation of upper extremity MSD has been described by other studies. A higher prevalence of shoulder pain among women versus men has also been observed in other health professions in China, such as dentists (OR 1.96) [9] and other non-health professions, such as office workers (OR 2.25) [45]. Silverstein et al. [5] and Armstrong et al. [46] demonstrated that women tend to be at higher risk for hand/wrist pain or injury when performing the same job as men. The prevalence of carpal tunnel syndrome in women may be up to 3 times higher than in men [47]. This could be explained by differences in anthropometric measures, muscle strength, or other sex-specific characteristics of the upper extremity between men and women [47–48]. Muscle strength is correlated with the prevalence of musculoskeletal pain due to the difference in endurance of heavy workloads [49]. The design of ultrasound equipment, tools, and workstations should take special consideration of the characteristics of women since they make up the majority of sonographers.

In this study, psychological factors (psychological fatigue and mental stress in the work place and self-perceived health status) were found to be associated with musculoskeletal pain. Sonographers who experienced a higher level (vs. a lower level) of psychological fatigue seemed more likely to present with neck pain. Shoulder and elbow pain were more likely to present in sonographers of “average” health status compared with those of “good” health status. A higher proportion of sonographers who indicated a high level of mental stress reported shoulder pain. The effect of psychological factors on the presentation of musculoskeletal pain and discomfort is supported by other studies [50–53]. Eatough et al. [51] suggested an association between psychological strain and a higher presentation of MSD symptoms in the wrist/hand, shoulder, and lower back among full-time employees mostly from retail/service (e.g., customer service representative) and professional industries (e.g., nurses). This association can potentially be explained by the tense scheduling, the stress of fast and accurate diagnosing, and high job demands. One of the effective interventions to relieve job stress and fatigue is job design, as both job design and environmental stressors are important factors contributing to employee health.

The number of scans performed per day is an important workload parameter. This was demonstrated to be a risk factor for lower back pain. Sonographers who scan >50 patients per day had 3.6 times greater odds of experiencing lower back pain than those who scan <30 patients per day. The increase in the number of scans performed may increase workload, prolong sitting time, and lead to insufficient rest or breaks between patients. Our results contrasted with those of Schoenfeld [54], who demonstrated direct associations between both the number of scans performed per month and the scan time per patient with the prevalence of MSD. However, in our study, scan time showed only a weak negative association with lower back pain in the univariate analysis (<10 minutes vs. >10 minutes, OR 0.49). The negative association between scan duration and lower back pain may be explained as follows: scan duration per patient might decrease with increasing work experience, and a weak negative correlation was found between time required to perform a scan and work years in our study (ρ = -0.14, P = 0.033). In the current study, sonographers reported an average of 8.7 hours per workday, of which 7.5 hours were spent on scanning. These hours are higher than the work hours reported by Pike et al. [15]. The number of scans performed per day (42.2% participants scan over 50 patients per day) in our study was much higher than in other studies, but the minutes spent per patient scan were much shorter than studies from other countries [15–16, 55]. With increasing hours spent scanning per week, wrist/hand pain increased significantly in the univariate analysis. This is probably because longer scan hours mean a longer period of maintaining awkward wrist/hand postures, repetition, and high-pressure force. The results of our study demonstrated the workload (scan hours and the number of scans per day) of Chinese sonographers was much heavier than that of sonographers in Western countries. Considering the difference in number of visits and inpatients among different level of hospitals, the workload in this study may be higher than that of other levels of hospital care in China. Measures should be taken to limit the maximum number of scheduled exams for sonographers to control the exposure to risk factors.

Scanning obese patients had effects on wrist/hand pain. Respondents who scanned more obese patients reported odds 2.6 times higher than those who scanned fewer obese patients. Scanning obese patients likely requires higher pressure force and the need to adopt uncomfortable postures to achieve diagnostic images [2]. These factors have been demonstrated to be significantly associated with wrist/hand pain and carpal tunnel syndrome [54, 56]. We also found exerting force with arm had positive effect on neck, shoulder, wrist/hand, and lower back pain in the univariate analysis. One way to make it easier to scan obese patients is to use transducers that can image in wide soft tissue windows and at greater depths [57]. The problem of scanning obese patients and exerting big pressure force might be solved in the future with the development of improved technology and transducers.

Repetitive movements and prolonged working postures have been shown to be related to MSD in sonographers [58]. Awkward working postures are a risk for strain injury and have been demonstrated by the current study to be significantly correlated with lower back pain. This is consistent with previous studies regarding occupational MSD [32, 59–61]. Bending or twisting the neck showed no significant difference in neck pain in the final multivariate analysis, but did aggravate upper and lower back injuries in our study. Bending or twisting the neck might occur together with other postures, such as bending the trunk, sitting, and other trunk postures, which could be associated with back pain [32]. Sonographers who have to bend and twist the trunk for long periods were likely to develop neck and shoulder pain. Bending the trunk frequently significantly increased the physical damage to the lower back, which is in agreement with findings of a previous study where twisting and bending the trunk and bending the neck forward were risk factors for lower back pain in Chinese workers [32]. MSD in the shoulder region (shoulder and upper back) was associated with having to maintain shoulder abduction for long periods of time, consistent with results from Wihlidal and Kumar [41], and Russo et al. [36]. Bending the wrist was found to be risk factor for wrist/hand disorders (such as carpal tunnel syndrome) by Schoenfeld et al. [54]. In our study, frequent bending of the wrist was significantly associated with wrist/hand pain.

The study has produced results that will be of use in improving the ergonomics and comfort of sonographers’ workplaces in China. The results showed that sonographers in China are in urgent need of ergonomic work facilities and devices, especially height-adjustable tables and chairs. Height-adjustable tables were a protective factor for neck pain in our research, which contrasted with Claes’s finding that height-adjustable tables increased the occurrence of neck pain [35]. However, we found that height-adjustable tables were a risk factor for elbow pain. Height-adjustable chairs could reduce the risk of neck, shoulder, and upper back pain in our study, whilst chairs with footrest could decrease the risk of suffering lower back pain. In fact, poor ergonomic designs of tables, chairs, and equipment are important contributing factors for developing MSD. We conducted a further analysis to determine the effect of adjustable chairs and tables on working postures. Height-adjustable tables had a significant effect on awkward postures (OR 4.3) and bending and twisting the trunk (OR 4.1). Height-adjustable chairs showed significant effect on the posture of bending neck backward (nonadjustable VS. adjustable, OR 1.9, 95% CI 1.07–3.50). Height-adjustable tilt tables (for special scan procedures), movable tables, and height-adjustable chairs are highly recommended by sonographer/sonologist associations and ergonomic researchers [39, 62–63] to ensure an upright posture and optimize the position with the patient when necessary. In this study, only 9.9% reported having height-adjustable tables, and 40.5% reported the chairs they used were non-adjustable. The percentage of adjustable tables and chairs used was very low compared to the same profession in other Western countries [16, 35]. It can be inferred that the adjustability of tables and chairs in primary and secondary hospitals might be even poorer because of the higher cost of ergonomic facilities. Managers of hospitals or the department of medical ultrasound should take these findings into account when choosing tables and chairs for sonographers.

The location of ultrasound devices had a significant effect on the presentation of upper back pain, with positioning in front of the sonographer being protective. Being aware of adjusting the workstation before scanning reduced the risk of shoulder pain in our study. Adjusting the workstation before starting work may help to protect sonographers from awkward working postures. However, in our study only 22.4% of the respondents were aware of the need to adjust the workstation. We also analyzed the association between the awareness and the posture complaints. The results show that awareness had significant effect complaints related to trunk twisting (OR 1.9; 95% CI, 1.01–3.63) and bending the neck backward (OR 2.8; 95% CI, 1.18–6.55). Thus, it is important to raise the awareness of adjusting workstations to maintain better working postures. A comparable rate of ergonomic awareness (15.4%) was also reported by Liang et al. among urologic surgeons in China [13]. This indicates the need of ergonomic training and MSD preventive guidelines for sonographers in China.

Our study demonstrated that the frequently used ultrasound devices were mostly imported from Europe and the USA, which are consistent with a previous survey [22]. Inappropriate design of the height of control panel and screen, and the transducer size may result in uncomfortable work postures, and increase the likelihood of developing MSD [35, 64–65]. Overall, 40.9 percent of participants in this study reported that the screen was higher than their eye level, and more than half of the participants indicated that the design of equipment/transducers needs to be improved. However, no significant differences were found in the presentation of MSD between ultrasound devices produced internationally or domestically. This can be interpreted as the ergonomics of ultrasound devices is not isolated, but includes other elements like chairs, tables, devices, workspace, and people in the system. Hence, we should adopt a systemic view when designing the work systems. In addition, it can be inferred that ultrasound devices used in China lack ergonomic design, regardless of whether they were produced domestically or internationally, because of the lack of anthropometric data, ergonomic knowledge, and practical use in industry in China. The anthropometric data of Chinese adults used nowadays were published in 1988 [66] and are outdated. Therefore, sonographers in China may face the problem of device ergonomics no matter which level of hospitals they work in and which devices they use. Poorly designed facilities may have negative effects on patients, and scanning under poor ergonomic conditions might increase sonographer fatigue and cause diagnostic error. Further studies are needed to investigate the association between the design of the devices and facilities with the musculoskeletal symptoms so as to help Chinese sonographers design appropriate work devices and facilities.

Overall, the results of the current study demonstrated a high prevalence of musculoskeletal symptoms in different body sites in sonographers from tertiary hospitals in central China. The participants in this study were sampled from 14 randomly selected tertiary-level hospitals of different cities in Hubei province, which improves the generalizability of the results for sonographers working in tertiary hospitals in China. The prevalence of MSD was affected by demographic and psychosocial factors, especially working postures and the ergonomics of work facilities and devices. We should take measures to improve the whole work system design since the factors contributing to MSD are complex and interact with each other. Future studies should explore concrete and effective intervention strategies. Our study revealed serious ergonomic problems related to working facilities and devices. These are generalizable to the average sonographer in China, given the general lack of ergonomic design and anthropometric data in China. Although these results cannot be extrapolated directly to all Asian populations, they can provide valuable references for other Asian countries to highlight the need to improve sonographers’ workplace ergonomics. It would be valuable if detailed measurement data could have been collected, in terms of both the dimension and size of facilities and devices and anthropometry, to investigate whether the designs conform to the Chinese population. Sonographers and medical administrators in China need to raise awareness of risk factors through training and education programs, and to develop self-protective actions (such as adjusting chair, table, and workstation before starting work, and using ergonomically facilities and devices). Further research is needed, including on-site measurement of the physical dimensions of the equipment and work space, and establishing standards on ultrasound device and work space design for Chinese people.

## Limitations

It should be noted that one of the limitations of our study was sampling bias: only on-duty sonographers were studied during the survey procedure; thus, a large number who have retired, resigned, or who were absent (for example due to ill health) were not included in the sampling population. This means that important, valid data may have been missed. Another limitation is that our study ignored the quantitative interactions between psychological, physical, and personal factors. Further research needs to consider the relative relationships of intermediate factors to establish MSD development models for sonographers.

## Conclusions

This study revealed an extremely high prevalence of musculoskeletal pain in Chinese sonographers. Furthermore, it examined the contribution of risk factors for MSD according to different upper body regions. This has not been studied previously. Occupational injuries to different parts of the body are likely to be induced by multiple factors—including personal factors, work scheduling, heavy work load, working postures, and poorly designed facilities—that do not match the demographics of Chinese people. Our study revealed significant issues around equipment, facilities, and working postures that should be addressed accordingly. Further research into ergonomic on-site measurements of the identified predictors may help to identify the solutions to improve the current status. Furthermore, sonographers were generally found to lack awareness to protect them from musculoskeletal injury. There is urgent need for popularizing the principles of ergonomics, and establishing human factors and ergonomics training programs among healthcare professionals and medical students. We hope our research will raise awareness of the urgent need for ergonomic solutions for sonographers in China.

## Supporting Information

S1 Appendix Relevant data underlying the findings described in manuscript. (XLSX)

S1 STROBE Statement Checklist of items that should be included in reports of observational studies. (DOCX)
