# Supplementary material for: The Prevalence of and Risk Factors Associated with Musculoskeletal Disorders among Sonographers in Central China: A Cross-Sectional Study
Source: PLoS One. 2016 Oct 3;11(10):e0163903. doi: 10.1371/journal.pone.0163903 (PMC5047644; doi:10.1371/journal.pone.0163903)
Supplement: S1 STROBE Statement — (DOCX) [file pone.0163903.s002.docx]

STROBE Statement—checklist of items that should be included in reports of observational studies

|  | Item No. | Recommendation | Page  No. | Relevant text from manuscript |
| --- | --- | --- | --- | --- |
| **Title and abstract** | 1 | (*a*) Indicate the study’s design with a commonly used term in the title or the abstract | 1 | A Cross-Sectional Study |
|  |  | (*b*) Provide in the abstract an informative and balanced summary of what was done and what was found | 2 | Self-reported questionnaire; 381 sonographers of 14 randomly selected tertiary hospitals; Multivariate logistic regression; high prevalence of MSD in different body sites were found, with physical workload, and equipment/facility-related factors and psychological as associated risk factors. |
| Introduction | | | |  |
| Background/rationale | 2 | Explain the scientific background and rationale for the investigation being reported | 4-6 | The prevalence of and associated risk factors among Chinese sonographers have not been evaluated. The workload and the workplace ergonomics of sonographers in China are different from their western colleagues. |
| Objectives | 3 | State specific objectives, including any prespecified hypotheses | 6 | Investigate the prevalence and severity of MSD and identify the associated risk factors in various body sites. |
| Methods | | | |  |
| Study design | 4 | Present key elements of study design early in the paper | 6 | Sonographers working in tertiary hospitals; started in March 2015, planned to complete within four months. |
| Setting | 5 | Describe the setting, locations, and relevant dates, including periods of recruitment, exposure, follow-up, and data collection | 6-7 | Study duration: March 2015-July 2015; tertiary hospitals in Hubei province; collect each posted questionnaire within 4 weeks. |
| Participants | 6 | (*a*) *Cohort study*—Give the eligibility criteria, and the sources and methods of selection of participants. Describe methods of follow-up  *Case-control study*—Give the eligibility criteria, and the sources and methods of case ascertainment and control selection. Give the rationale for the choice of cases and controls  *Cross-sectional study*—Give the eligibility criteria, and the sources and methods of selection of participants | 7 | Sonographers working in tertiary hospitals in Hubei province, with at least 1 year work experience were invited; sonographers with pain or discomfort caused by trauma or accidents were excluded; |
|  |  | (*b*) *Cohort study*—For matched studies, give matching criteria and number of exposed and unexposed  *Case-control study*—For matched studies, give matching criteria and the number of controls per case |  |  |
| Variables | 7 | Clearly define all outcomes, exposures, predictors, potential confounders, and effect modifiers. Give diagnostic criteria, if applicable | 7-8 | Outcomes: Musculoskeletal pain or discomfort; exposures: demographic and psychosocial factors, work scheduling and tasks, working posture, and work equipment, facility; potential confounding factors: age, gender, education |
| Data sources/ measurement | 8* | For each variable of interest, give sources of data and details of methods of assessment (measurement). Describe comparability of assessment methods if there is more than one group |  |  |
| Bias | 9 | Describe any efforts to address potential sources of bias | 9 | Spearman correlation matrix was used to diagnose collinearity; Multivariate logistic regressions were used to determine the effects of potential risk factors on MSD symptoms with crude and adjusted OR (adjusted for gender, age and education). |
| Study size | 10 | Explain how the study size was arrived at | 6 | Based on sample size calculations, using an estimated 80% prevalence of MSD and allowing a 5% tolerable error, based on a 95% confidence interval (CI).~2,000 population. |

Continued on next page

| Quantitative variables | 11 | Explain how quantitative variables were handled in the analyses. If applicable, describe which groupings were chosen and why | 9 | Continuous variables were divided into three groups using the tertile boundaries. |
| --- | --- | --- | --- | --- |
| Statistical methods | 12 | (*a*) Describe all statistical methods, including those used to control for confounding | 9 | Descriptive statistics; univariate logistic regression ; Spearman correlation matrix; Multivariate logistic regressions were used to determine the effects of potential risk factors on MSD symptoms with crude and adjusted OR (adjusted for gender, age and education). |
|  |  | (*b*) Describe any methods used to examine subgroups and interactions | 9 | Spearman correlation matrix was used to diagnose collinearity; |
|  |  | (*c*) Explain how missing data were addressed | 10 | Over 20% missing items were excluded analysis. Other missing items were defined as system-missing values. |
|  |  | (*d*) *Cohort study*—If applicable, explain how loss to follow-up was addressed  *Case-control study*—If applicable, explain how matching of cases and controls was addressed  *Cross-sectional study*—If applicable, describe analytical methods taking account of sampling strategy |  |  |
|  |  | (*e*) Describe any sensitivity analyses |  |  |
| Results | | | | |
| Participants | 13* | (a) Report numbers of individuals at each stage of study—eg numbers potentially eligible, examined for eligibility, confirmed eligible, included in the study, completing follow-up, and analysed | 11 | Table 1 |
|  |  | (b) Give reasons for non-participation at each stage |  |  |
|  |  | (c) Consider use of a flow diagram |  |  |
| Descriptive data | 14* | (a) Give characteristics of study participants (eg demographic, clinical, social) and information on exposures and potential confounders | 11 | Table 1 |
|  |  | (b) Indicate number of participants with missing data for each variable of interest | 11-14 | Tables 1-3 |
|  |  | (c) *Cohort study*—Summarise follow-up time (eg, average and total amount) |  |  |
| Outcome data | 15* | *Cohort study*—Report numbers of outcome events or summary measures over time |  |  |
|  |  | *Case-control study—*Report numbers in each exposure category, or summary measures of exposure |  |  |
|  |  | *Cross-sectional study—*Report numbers of outcome events or summary measures | 16 | Table 4 |
| Main results | 16 | (*a*) Give unadjusted estimates and, if applicable, confounder-adjusted estimates and their precision (eg, 95% confidence interval). Make clear which confounders were adjusted for and why they were included | 18-21 | Tables 5-10. Adjusted by age, sex, and education. |
|  |  | (*b*) Report category boundaries when continuous variables were categorized | 11-14 | Tables 1-3 |
|  |  | (*c*) If relevant, consider translating estimates of relative risk into absolute risk for a meaningful time period |  |  |

Continued on next page

| Other analyses | 17 | Report other analyses done—eg analyses of subgroups and interactions, and sensitivity analyses |  |  |
| --- | --- | --- | --- | --- |
| Discussion | | | | |
| Key results | 18 | Summarise key results with reference to study objectives | 22 | 98.3% of respondents reported scanning whilst in pain or discomfort in the past 12 months. Work related MSDs were most frequently reported in the neck, shoulder, lower back, and wrist/hand regions. Individual characteristics, psychological factors, working postures, and work facilities were shown to contribute to the prevalence of MSDs in the four regions of upper body. |
| Limitations | 19 | Discuss limitations of the study, taking into account sources of potential bias or imprecision. Discuss both direction and magnitude of any potential bias | 29-30 | Sampling bias; ignored quantitative interactions between independent variables. |
| Interpretation | 20 | Give a cautious overall interpretation of results considering objectives, limitations, multiplicity of analyses, results from similar studies, and other relevant evidence | 22-30 |  |
| Generalisability | 21 | Discuss the generalisability (external validity) of the study results | 29 |  |
| Other information | |  | | |
| Funding | 22 | Give the source of funding and the role of the funders for the present study and, if applicable, for the original study on which the present article is based |  |  |

*Give information separately for cases and controls in case-control studies and, if applicable, for exposed and unexposed groups in cohort and cross-sectional studies.

**Note:** An Explanation and Elaboration article discusses each checklist item and gives methodological background and published examples of transparent reporting. The STROBE checklist is best used in conjunction with this article (freely available on the Web sites of PLoS Medicine at http://www.plosmedicine.org/, Annals of Internal Medicine at http://www.annals.org/, and Epidemiology at http://www.epidem.com/). Information on the STROBE Initiative is available at www.strobe-statement.org.
